# Impact of sub-thalamic nucleus deep brain stimulation on dual tasking gait in Parkinson’s disease

**DOI:** 10.1186/1743-0003-10-38

**Published:** 2013-04-15

**Authors:** Eliraz Seri-Fainshtat, Zvi Israel, Aner Weiss, Jeffrey M Hausdorff

**Affiliations:** 1 Department of Neurosurgery, Center for Functional & Restorative Neurosurgery, Hadassah University Hospital, Jerusalem, Israel; 2 Department of Physical Therapy, Sackler Faculty of Medicine and Sagol School of Neuroscience, Tel-Aviv University, Tel-Aviv, Israel; 3 Movement Disorders Unit, Tel-Aviv Sourasky Medical Center, Tel-Aviv, Israel; 4 Department of Medicine, Harvard Medical School, Boston, MA, USA

**Keywords:** Dual task, Falls, Executive control, Parkinson’s disease, Cognitive impairment, Gait, DBS

## Abstract

**Background:**

The beneficial effects of bilateral sub-thalamic nucleus deep brain stimulation on motor function and gait in advanced Parkinson’s disease are established. Less is known about the effect of stimulation on cognitive function and the capacity to walk while dual tasking, an ability that has been related to fall risk. Everyday walking takes place in complex environments that often require multi-tasking. Hence, dual tasking gait performance reflects everyday ambulation as well as gait automaticity. The purpose of this study was to examine the impact of sub-thalamic nucleus deep brain stimulation on dual task walking in patients with advanced Parkinson’s disease.

**Methods:**

Gait was assessed using a performance-based test and by quantifying single-task and dual task walking conditions in 28 patients with advanced Parkinson’s disease. These tests were conducted in 4 conditions: “OFF” medication, with the stimulator turned on and off, and “ON” medication, with the stimulator turned on and off. A previously validated, computerized neuro-psychological battery assessed executive function, attention and memory “OFF” and “ON” deep brain stimulation, after subjects took their anti-Parkinsonian medications.

**Results:**

Stimulation improved motor function and the spatiotemporal parameters of gait (e.g., gait speed) during both single-task and dual task walking conditions. Attention improved, but executive function did not. The dual task effect on gait did not change in response to stimulation. For example, during serial 3 subtractions, gait speed was reduced by -0.20 ± 0.14 m/sec while OFF DBS and OFF meds and by -0.22 ± 0.14 m/sec when the DBS was turned on (p = 0.648). Similarly, ON medication, serial 3 subtractions reduced gait speed by -0.20 ± 0.16 m/sec OFF DBS and by -0.22 ± 0.09 m/sec ON DBS (p = 0.543).

**Conclusions:**

Bilateral sub-thalamic nucleus deep brain stimulation improves motor symptoms, certain features of gait and even some aspects of cognitive function. However, stimulation apparently fails to reduce the negative impact of a dual task on walking abilities. These findings provide new insight into the effects of deep brain stimulation on gait during cognitively challenging conditions and everyday walking.

## Background

In patients with advanced Parkinson’s disease, sub-thalamic nucleus (STN) deep brain stimulation (DBS) reduces tremor, rigidity and bradykinesia, allows for a significant decrease in anti-Parkinsonian medications, and diminishes dyskinesias [1-4]. Indeed, pre-surgery Unified Parkinson’s Disease Rating Scale (UPDRS) motor scores may often be improved by two-thirds in response to DBS [2-5]. DBS also enhances postural control and increases gait speed and stride length, both off and on anti-Parkinsonian medications [6-9]. From this perspective, DBS appears to provide good relief for key motor symptoms of advanced PD.

The effects of DBS on the ability to walk while performing a dual task (DT) have not been previously studied. Everyday walking typically takes place in complex environments and challenging situations that demand appropriate allocation of attention between gait and simultaneously-performed additional tasks such as talking on a cellphone [10,11]. Thus, whereas single-task walking represents the capabilities of a patient, DT walking abilities more closely reflect functional ambulation in cognitively-demanding conditions. DT walking abilities also provide a window into the automaticity of movement [10,12]. Over-learned movements do not depend on attention or cognitive input and are generally impervious to the effects of dual tasking. For example, healthy young adults can walk and perform other tasks with little impact on their gait. Conversely, when gait is impaired or relies on cognitive input, a DT will cause marked changes in the walking pattern. The simultaneous performance of a DT generally causes patients with PD to walk more slowly, with shorter strides, and with a much larger effect of the DT on gait than seen in healthy controls [11,13-16]. Pilot studies have demonstrated that interventions may enhance DT abilities even in PD [17-20], however, the effects of STN DBS on DT gait have not yet been investigated.

A priori, there are several ways to view the impact of DBS on DT walking. One possibility is that the negative DT effects, i.e., the DT costs, might be reduced as a result of DBS due to the improved motor function. If walking becomes a more automatic process after DBS and walking capacity improves, then it should be more resistant and impervious to the negative effects of DT. In other words, DBS should improve functional reserve so that it is less vulnerable to the effects of a DT. Alternatively, although gait improvement does occur in response to DBS, not all aspects of gait respond similarly to DBS [21-25]; for example, freezing of gait typically does not respond well to traditional STN DBS [26]. From this perspective, perhaps those gait properties that are more critical to DT abilities will not be enhanced. The effects of DBS on cognitive function are inconsistent, with some studies even reporting a decline in executive function following DBS [2,27,28]. Since DT abilities are also related to cognitive abilities, especially executive function [10,29], it could be speculated that DBS might not ameliorate the DT costs.

The primary purpose of the present study was to address these questions by quantifying the DT effects on gait in patients with PD while ON and OFF DBS. We hypothesized that STN DBS (ON DBS) reduces the DT costs of gait, compared to the costs observed when the stimulator is off (OFF DBS). To investigate the effects of DBS in a state that more closely reflects the underlying pathology, we examined the subjects during the OFF medication state. To evaluate conditions that reflect everyday function, patients were also assessed in the ON state (ON, OFF meds and ON and OFF DBS). Analysis focused on two properties of gait: 1) average stride length (and the closely related average gait speed). Stride length and gait speed are widely accepted measures of mobility in general [30], and in PD specifically [31,32]. 2) measures of gait variability. These gait features reflect the rhythmicity and consistency of the gait pattern, are altered in PD, are related to disease severity, and are related to fall risk [15,33-37]. Because of the potential role of cognitive function in DT costs, we also quantified the effects of DBS on this domain to gain insight into the relationship between the STN, cognitive function, and DT abilities in patients with advanced PD.

## Methods

### Participants

Subjects were included if they fulfilled UK Brain Bank criteria for PD [38], if they had undergone bilateral STN DBS surgery at least two months prior to the study for treatment of PD symptoms, if their stimulation settings were stable, and if they could walk independently without any walking aids, with the DBS on. Standard clinical criteria were used to evaluate the subjects for their suitability for STN DBS surgery. Selection criteria that were applied are those that have become widely accepted in many parts of the world. This includes, for example, at least a 30% improvement in UPDRS scores in response to an l-dopa challenge; a diagnosis of idiopathic PD for at least 4 or more years; complications of medication including ON-OFF fluctuations and/or dyskinesias; normal brain MRI; and otherwise generally healthy. Psychiatric exclusions include sub-optimally controlled depression. Psychotic patients are automatically excluded. To be approved for DBS surgery, the patients undergo a battery of cognitive tests. Addenbrooke’s Cognitive Examination (ACE) is used as a cognitive screen (subjects are included if they score over 70), along with the Frontal Assessment Battery (FAB), for which we generally will require a score of at least 12. The Hamilton Depression Rating Scale (HDRS) is used to screen for depression; subjects who score more than 15 will be excluded or sent for more detailed evaluation. Frank dementia is obviously an exclusion factor. Between 50% and 70% of patients who are referred to our center for surgery meet accepted inclusion criteria. The institutional review board (IRB) of Hadassah University Hospital approved this study and informed written consent was obtained.

### Protocol

Subjects arrived for the assessment in the morning, at least 12 hours after taking their last anti-Parkinsonian medication dose, i.e., in an OFF meds/ON DBS state, when medication effects are minimal [39,40]. While OFF medications, they were familiarized with the gait and dual tasks. Subjects then completed gait testing, the Timed Up and Go test, and the motor part of the UPDRS, both ON and OFF DBS. Subjects then took their usual morning dose of anti-Parkinsonian medications. After reaching a self-declared ON medication state (typically 30–60 minutes later), gait, the Timed Up and Go, and the motor part of the UPDRS were re-evaluated. A neuropsychological battery quantified cognitive function both ON and OFF DBS, while ON meds. The order of testing (ON vs. OFF DBS), was randomized both while OFF medications and while ON medications. With ample breaks, the entire protocol typically took about 3 hours to complete.

#### Gait tasks

Gait was studied in three conditions: 1) “single-task”, usual-walking at a self-selected comfortable speed (ST walking), 2) walking while subtracting serial 3s from a predefined 3 digit number (DT-S3) and 3) walking while performing a verbal fluency task (DT-VF) (without explicit instructions regarding prioritization); they were asked to recite words that began with a specific letter (the letter was randomized across conditions), using a previously validated protocol [41,42]. To determine if the effects of DBS on DT gait were dependent on the specifics of the dual task, both the arithmetic and fluency tasks were used. The order of testing (ST, DT-S3, and DT-VF) was randomized. Under each condition, subjects walked up and down an 18 meter-long, 2-meter wide hallway at their self-selected speed for one minute.

#### Secondary tasks

In each medication/DBS condition, serial subtraction (using a different 3 digit starting number) was also performed for one minute in the seated position to allow for quantification of the effects of walking on these cognitive tasks. The order of testing, i.e., seated vs. walking, was randomized. To minimize testing time, verbal fluency was not tested in the seated position. For both secondary tasks, the number of errors and number of words or subtractions were scored to evaluate performance on the serial subtraction and verbal fluency tests.

For each walking condition, several gait measures were derived from force sensitive shoe insoles and a stopwatch, using previously described and validated methods [14,15,19]. Briefly, steady-state gait speed was calculated by measuring the time to complete the middle 10 meters of each lap, averaged over all laps for each walk. Additional gait parameters that were evaluated included: average stride length, average stride time, average swing time, and gait variability, as measured by stride time variability and swing time variability [14,15,19]. Stride time was defined as the time from initial foot contact to the next initial foot contact. Swing time, presented as a percent of the gait cycle, was defined as the time of single support; it is determined by the time from toe-off to heel-strike, divided by the stride time (×100) and presented as a percent of the gait cycle (for healthy young adults, swing time % is about 40% of the gait cycle). Variability was defined using the coefficient of variation, e.g., 100 × (standard deviation of stride time)/(average stride time). Using previously described methods, the data at each turn (that took place when the subject reached the end of a lap) and any data during freezing of gait episodes, were excluded from the analysis. Any freezing of gait episode was counted in each walking condition. To compare the DT effects across DBS and medication states, the DT cost measure was calculated as the difference between the single-task and DT walking conditions, for each gait parameter [10,11]. We also verified that the results were similar if DT costs were calculated as percent change instead of the difference. Since both methods of estimating DT costs produced similar findings, only the results based on the differences are reported.

### Assessment of PD symptoms and cognitive function

PD status and disease severity were evaluated using the motor portion of the UPDRS [43]. Mean L-dopa daily dose was quantified as previously described [44]. The Timed Up and Go test assessed functional mobility [45,46] (standard instructions were followed; there was no dual task during this test). A computerized neuropsychological test battery (Mindstreams®, NeuroTrax Corp., NJ), previously validated in patients with PD [15,47] quantified executive function, memory and attention (largely sustained attention) [48,49]. The executive function battery included computerized versions of the Go-No-Go and the Stroop interference tests. The test battery generates composite indices of each cognitive domain [48-50] on an IQ-like scale, with 100 representing the estimated population mean normalized for age and education.

### Statistical analysis

Descriptive statistics are reported as mean ± standard deviation. Many of the dependent variables were not normally distributed; hence, non-parametric analyses were applied since they make no assumptions about the distribution of the data and are not sensitive to extreme values. To assess the effects of DBS on gait, Friedman’s tests (the non-parametric parallel to repeated measures ANOVA) were first applied for each gait parameter (e.g., gait speed) to determine if any of the walking conditions differed from one another (i.e., ST, DT-S3, DT-VF). If differences were observed (p < 0.05), Wilcoxon Signed Rank tests were applied to determine which conditions differed. Similar analyses were applied to the cognitive measures, other patient characteristics, and to the DT costs. P-values reported are based on two-tailed comparisons. The significance level was set at 0.05. Statistical analyses were performed using SPSS for Windows.

## Results

### Subject characteristics

In a consecutive sample spanning from October 2007 to November 2008, 28 patients with PD met the inclusion and exclusion criteria and agreed to participate. Patient characteristics are summarized in Table 1 and Table 2. Stimulation parameters included a mean rate of 161.96 ± 24.01 Hz for the left or the right side. The mean voltage was 3.08 ± 0.58 V for the right side and 3.12 ± 0.61 V for the left side. Pulse width was 90 μs for 10 electrodes (in 6 patients) and 60 μs for the other 46 electrodes. Mean L-dopa daily dose was 1180 ± 758 mg before surgery and 490 ± 269 mg after surgery (p < 0.001); with a median decrease in medication dose of 60% (Dopa-equivalent).

**Table 1:** Subject characteristics (n = 28)

**Age (years)**	61.46 ± 8.13
**Gender**	3 females
**Disease duration (years)**	13.2 ± 5.0
**Mini Mental State Exam**	27.9 ± 1.7
**Time after surgery (months)**	25.4 ± 13.1
**UPDRS motor scores (OFF DBS/OFF meds)**	38.53 ± 13.53

**Table 2:** Effects of DBS on motor symptoms and single-task, usual-walking gait

	**OFF meds**			**ON meds**		
	**OFF DBS**	**ON DBS**	**P-value OFF vs. ON DBS**	**OFF DBS**	**ON DBS**	**P-value OFF vs. ON DBS**
**UPDRS Motor part**	38.53 ± 13.53	17.5 ± 8.20	<0.001	36.20 ± 13.84	13.40 ± 7.82*	<0.001
**Timed Up and Go (sec)**	14.97 ± 5.69	12.62 ± 4.42	0.001	13.28 ± 3.27	11.46 ± 2.35*	0.007
**Gait speed (m/s)**	1.01 ± 0.30	1.09 ± 0.31	0.009	1.13 ± 0.19	1.21 ± 0.18*	0.053
**Stride length (m)**	1.09 ± 0.29	1.16 ± 0.30	0.005	1.27 ± 0.14	1.32 ± 0.15*	0.049
**Stride time (sec)**	1.10 ± 0.12	1.090 ± .10	0.502	1.09 ± 0.10	1.09 ± 0.10	0.653
**Stride time variability (%)**	2.82 ± 1.42	2.33 ± 1.24	0.093	2.19 ± 0.91	1.84 ± 0.46*	0.407
**Swing time (%)**	36.19 ± 3.09	36.18 ± 2.68	0.654	36.66 ± 2.87	36.90 ± 2.05	0.199
**Swing time variability (%)**	5.35 ± 3.10	4.65 ± 2.81	0.156	4.68 ± 2.02	3.94 ± 1.20	0.035

*indicates a significant difference between OFF meds/OFF DBS vs. ON meds/ON DBS.

### Effects of DBS on motor function and single-task walking

UPDRS motor scores improved significantly (p < 0.0001) with DBS stimulation (both ON and OFF meds), and when comparing the two extreme states (i.e., OFF meds/OFF DBS vs. ON meds/ON DBS) as seen in Table 2. Similarly, Timed Up and Go times decreased (i.e., improved) in response to DBS, both ON meds (p = 0.007) and OFF meds (p = 0.001). Timed Up and Go duration in the ON meds/ON DBS condition was significantly lower than that in the OFF meds/OFF DBS condition.

Table 2 summarizes the single-task walking parameters in the 4 medication/DBS conditions. Gait speed improved in response to DBS in the OFF meds condition (p = 0.009). In the ON meds condition, gait speed also tended to improve, but this increase was not significant (p = 0.053). Gait speed and stride length in the ON meds/ON DBS condition were significantly greater than those in the OFF meds/OFF DBS condition. Stride time variability improved (i.e., decreased) in response to DBS in the OFF meds condition, however, this change did not reach the level of significance (p = 0.093). Swing time variability significantly improved in response to DBS in the ON meds condition (p = 0.035).

### Effects of DBS on DT costs

Both dual tasks (serial subtractions and verbal fluency) had a significant negative impact on almost all gait parameters in all 4 conditions, compared to single-task walking (typically p < 0.0001 under every condition, for all gait parameters). For example, in the OFF meds/OFF DBS condition, gait speed was reduced from 1.01 ± 0.30 m/sec during single-task walking to 0.84 ± 0.29 m/sec (p < 0.0001) during serial 3 subtractions, and to 0.82 ± 0.28 m/sec (p < 0.0001) during the verbal fluency task. In the ON meds/ON DBS condition, gait speed was reduced from 1.08 ± 0.32 m/sec during single-task walking to 0.91 ± 0.29 m/sec during serial 3 subtractions (p < 0.0001), and to 0.87 ± 0.30 m/sec during the verbal fluency task (p < 0.0001).

**Figure 1:**
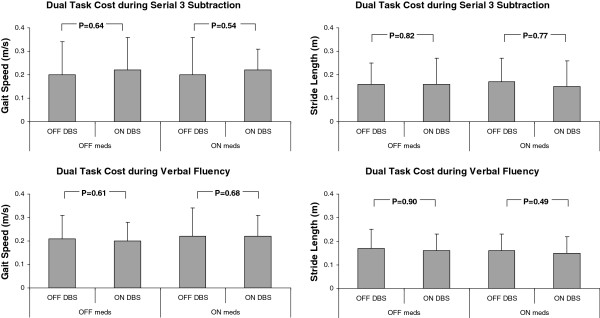
**The effects of DBS on dual costs on gait speed (left) and stride length (right) for the serial 3 subtraction (above) and verbal fluency tasks (below).** For visual purposes, costs are displayed as a positive number (i.e., dual task value – single-task value). Note that if these analyses were repeated after stratifying by time since surgery, the findings were essentially unchanged; for example, among the subjects who more than 12 months after surgery, there were no changes in DT costs (p > 0.463).

As summarized in Tables 3 and 4, the DT cost of all gait parameters did not change with stimulation compared to the DT cost without stimulation, when subjects were ON or OFF medications (see also Figure 1). The DT costs in the two extreme conditions (i.e., OFF meds/OFF DBS and the ON meds/ON DBS) were also not significantly different from each other for all gait parameters. As noted in the Methods, the lack of any beneficial effect of DBS on DT costs was also observed if costs were determined as percent change. For example, for gait speed, the percent change in each of the 4 conditions, corresponding to those in Table 3, were: -20.67% and -23.20% (P = 0.848, DBS effect OFF meds) and -16.38% and -20.20% (P = 0.778, DBS effect ON meds) during serial subtractions. Table 4 summarizes the effects of DBS on dual costs during the verbal fluency task. As was the case for serial subtraction, the DT cost of all gait parameters did not change with stimulation compared to the DT cost without stimulation, during the verbal fluency task.

**Table 3:** Effects of DBS on dual task costs during serial 3 subtractions*

	**OFF meds**			**ON meds**		
	**OFF DBS**	**ON DBS**	**P-value OFF vs. ON DBS**	**OFF DBS**	**ON DBS**	**P-value OFF vs. ON DBS**
**Gait speed (m/sec)**	-0.20 ± 0.14	-0.22 ± 0.14	0.648	-0.20 ± 0.16	-0.22 ± 0.09	0.543
**Stride length (m)**	-0.16 ± 0.09	-0.16 ± 0.11	0.823	-0.17 ± 0.10	-0.15 ± 0.11	0.778
**Stride time (sec)**	0.08 ± 0.11	0.08 ± 0.10	0.433	0.07 ± 0.10	0.09 ± 0.10	0.398
**Stride time variability (%)**	0.62 ± 0.81	0.84 ± 0.95	0.067	2.09 ± 3.17	1.01 ± 0.91	0.243
**Swing time (%)**	-1.09 ± 1.07	-0.93 ± 0.91	0.247	-1.21 ± 1.63	-0.81 ± 1.00	0.904
**Swing time variability (%)**	1.70 ± 1.44	1.78 ± 1.48	0.502	2.33 ± 3.97	1.19 ± 1.62	0.355

*Entries are single-task - dual task values.

**Table 4:** Effects of DBS on dual costs during the verbal fluency task*

	**OFF meds**			**ON meds**		
	**OFF DBS**	**ON DBS**	**P-value OFF vs. ON DBS**	**OFF DBS**	**ON DBS**	**P-value OFF vs. ON DBS**
**Gait speed (m/sec)**	-0.21 ± 0.10	-0.20 ± 0.08	0.615	-0.22 ± 0.12	-0.22 ± 0.09	0.681
**Stride length (m)**	-0.17 ± 0.08	-0.16 ± 0.07	0.903	-0.16 ± 0.07	-0.15 ± 0.07	0.494
**Stride time (sec)**	0.10 ± 0.12	0.07 ± 0.07	0.181	0.10 ± 0.13	0.08 ± 0.10	0.520
**Stride time variability (%)**	0.61 ± 1.29	1.18 ± 1.73	0.192	1.61 ± 1.99	1.23 ± 1.46	0.687
**Swing time (%)**	-1.01 ± 1.20	-1.13 ± 1.03	0.689	-1.23 ± 1.30	-1.45 ± 2.66	0.658
**Swing time variability (%)**	1.82 ± 1.59	2.00 ± 2.44	0.958	2.60 ± 3.80	3.36 ± 8.63	0.573

*Entries are single-task - dual task values.

### Performance of secondary tasks

Tables 5 and 6 summarize the performance of the secondary tasks in all 4 walking conditions. Briefly, in response to DBS, the number of serial subtractions performed during walking was unchanged OFF medications (p = 0.796). ON medications, the number of serial subtractions increased (p = 0.036). The number of mistakes made was not related to medication or DBS condition (see Table 6). Verbal fluency performance was also independent of medication and DBS condition.

**Table 5:** Performance on secondary tasks: number of subtractions (S3) and verbal fluency (VF)

	**OFF meds**			ON meds*		
	**OFF DBS**	**ON DBS**	**P-value OFF vs. ON DBS**	**OFF DBS**	**ON DBS**	**P-value OFF vs. ON DBS**
**S3 Sitting**	16.09 ± 7.84	17.77 ± 8.23	0.055	16.77 ± 9.12	17.50 ± 8.56	0.830
**S3 Walking**	15.61 ± 7.77	15.66 ± 8.07	0.796	15.05 ± 7.75	17.94 ± 8.27	0.036
**P-Value Sitting vs. Walking**	0.659	0.001		0.157	0.311	
**VF Walking**	6.26 ± 3.14	6.47 ± 1.92	0.732	6.66 ± 2.19	6.77 ± 2.57	0.855

*Entries are the mean ± standard deviation number of words recited in each condition for verbal fluency and the mean ± standard deviation number of subtractions during the serial 3 subtraction task.

**Table 6:** Performance on secondary tasks: mistakes in the number of subtractions or while generating words

	**OFF meds**			**ON meds**		
	**OFF DBS**	**ON DBS**	**P-value OFF vs. ON DBS**	**OFF DBS**	**ON DBS**	**P-value OFF vs. ON DBS**
**S3 Sitting**	1.04 ± 1.09	0.90 ± 1.06	0.476	0.88 ± 1.13	0.94 ± 0.99	0.803
**S3 Walking**	1.33 ± 1.13	1.44 ± 1.38	0.750	1.31 ± 1.70	1.42 ± 1.64	0.677
**P-Value Sitting vs. Walking**	0.408	0.694		0.239	0.770	
**VF Walking**	0.52 ± 0.84	0.89 ± 0.87	0.109	0.27 ± 0.57	0.72 ± 1.12	0.190

S3: serial 3 subtractions; VF: verbal fluency.

### Effects of DBS on freezing of gait

Freezing of gait (FOG) was observed in 9 subjects. FOG occurred in 12% of the walking tasks. The vast majority of the freezing (79%) took place during the turns at the end of each lap, i.e., only about 3% of the walks were interrupted by FOG in the middle of a lap. The number of FOG episodes was not significantly affected by DBS. For example, in the OFF medication condition (p > 0.18 overall), 3 episodes occurred during usual walking, both ON and OFF DBS. 4 episodes took place during serial 3 subtractions OFF DBS and 6 occurred ON DBS. Two FOG episodes occurred during the verbal fluency dual task off DBS, while 4 occurred when DBS was on.

### Effects of DBS on cognitive function

**Figure 2:**
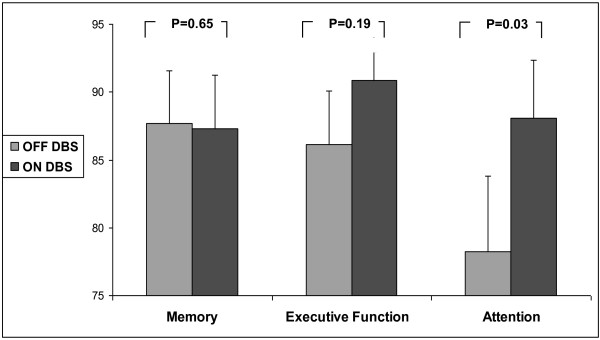
Differences in cognitive function index scores between ON and OFF DBS (while ON medications).

**Figure 3:**
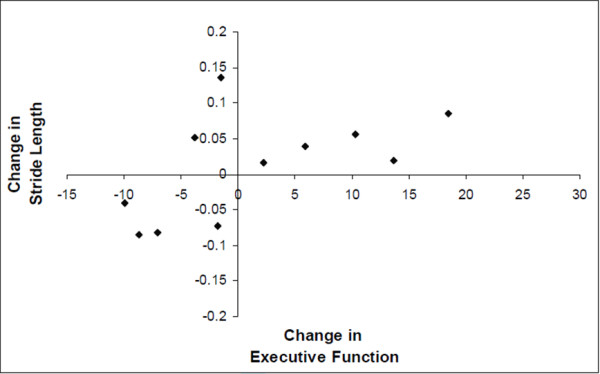
**Scatter plot showing the relationship between the change in executive function and change in DT stride length costs during the verbal fluency task in response to DBS (ON medications) (r = 0.65, p = 0.032).** In general, subjects who had better executive function scores in response to DBS also had lower DT costs (plotted as a positive change).

Figure 2 shows the cognitive index scores both ON and OFF DBS (while ON meds). Attention improved in response to DBS and executive function improved, however, the increase was not statistically significant. Memory did not improve in response to DBS. Interestingly, improvements in the executive function index in response to DBS were associated with lower (i.e., better) DT costs for stride length during the verbal fluency task (r = 0.65, p = 0.032) (see Figure 3). Similarly, improvements in the executive function index in response to DBS were associated with lower (i.e., better) DT costs for stride time variability during the serial 3 subtraction task (r = 0.66, p = 0.038).

## Discussion

STN DBS has previously been associated with improvements in motor function, stride length, gait speed and rhythmicity [6-9]. We, therefore, hypothesized that gait would be more consistent and automatic and less cognitively demanding when the stimulation was activated and that this effect would lead to a reduction in the DT costs of gait, compared to the costs observed when the stimulator is off. Our findings suggest that this hypothesis should be rejected. Although we found improvement in certain features of single-task gait and cognitive function with stimulation, the impact of DT on gait was unchanged in response to bilateral STN DBS.

One partial explanation might be that the effects of DBS on cognitive function were not sufficient or specific enough to elicit beneficial changes to DT walking. We found improvement in attention with STN stimulation, although executive function did not improve significantly. The attention index that we used largely reflects sustained attention, but not task switching or dual tasking. Executive function may be more closely associated with DT performance and complex everyday walking in PD [10,11,51]. While executive function did respond to DBS in some subjects and there was an association between improvement in this cognitive domain and DT costs (recall Figure 3), this finding was not consistently observed. Thus, this overall negative finding with respect to executive function might explain the lack of improvement in DT walking ability. Improvements in single-task gait alone are apparently not sufficient to lower the DT impact as some form of cognitive reserve is apparently involved during DT walking in patients with PD [12]. This observation that only specific sub-domains of cognitive function improved in response to DBS is consistent with previous studies [27,52]. Interestingly, Alberts et al. [53] reported that bilateral STN DBS leads to a decreased ability to track forces when the dual task was complex (e.g., serial 3 subtractions), somewhat parallel to the influence of serial subtractions on gait in the present study.

The basal ganglia have multiple connections to frontal networks that regulate executive function and cognitive abilities [54,55]. The present findings are consistent with previous studies that suggested that STN DBS does not positively influence these circuits and their function [2,27,28]. In addition to the basal ganglia-thalamocortical motor circuits, output from the basal ganglia also ends in the pedunculopontine nucleus (PPN), an area which has been termed the brain’s gait pace regulator [56-58]. Noting the ineffectiveness of STN DBS to reduce fall risk and freezing of gait, consistent with the present findings, several authors speculated that PPN stimulation may be more successful for ameliorating axial symptoms like freezing and falls [26,44,58]. In the future, it would be interesting to test whether DT costs respond to PPN stimulation.

### Clinical implications and limitations

Falls and freezing of gait, common causes of falls in PD, are, typically, unresponsive to STN DBS [21,28,58,59]. Weaver et al. even describe an increase in fall frequency post-DBS among patients with PD [60]. It is unclear if this increase is a direct effect of DBS or an indirect result [60]. Improved ambulation following DBS may lead to greater exposure to challenging situations and, hence, to an increased risk of falls. Among older adults and in patients with PD, many falls occur during ambulation and fall risk has been related to executive function, DT performance, gait variability, and usual-walking gait abilities [15,42,51,61-69]. In the present study, usual-walking gait speed improved in response to DBS, however, these other fall risk factors were generally not responsive to DBS. If subjects walk faster after DBS, while their cognitive function and ability to cope with challenging conditions are unchanged, as reflected in the DT costs, perhaps they have a greater risk for falls. In this regard, it is interesting to note that the present findings suggest that just as patients with PD inappropriately focus their attention on the secondary, dual tasks and apply a “posture second” strategy [12,16], this poor judgment is not responsive to DBS.

This study has several limitations. For example, the assessment protocol was long (approximately 3 hours) and some subjects became tired toward the end. This may have resulted in an under-estimation of the effects of anti-Parkinsonian medication, as the assessments with medication were always conducted last, when the subjects were most likely to be tired. On the other hand, this situation may actually reflect everyday abilities. Similarly, we did not assess the response to a supra-maximal dose of l-dopa, something that might be of interest in the future, because we aimed to focus on testing that reflects the subject’s typical abilities and medication dosing. We also did not make any statistical corrections for multiple comparisons. However, this analytical choice actually increased the likelihood that we might find support for lower DT costs in response to DBS; the results did not support this possibility, even though both dual tasks generally had robust (e.g., p < 0.001) negative effects on gait. Another limitation is that the assessor was not blinded to the condition. However, data analyses were performed offline using automated software routines that were blinded to the conditions. Finally, we did not include a healthy control group to compare the DT costs. However, previous studies reported higher DT costs among patients with PD compared to age-matched controls [11,15]. We, therefore, conjecture that the costs observed in the present study were also larger than those that could be expected to be seen in healthy controls.

## Conclusions

The present investigation is, to our knowledge, the first explicit examination of DT walking in PD patients after STN DBS. The DT costs of multiple, distinct aspects of gait did not improve in response to STN DBS. The absence of change in this ability with STN stimulation indicates that this surgery does not positively impact on the automaticity of walking and suggests that PD patients may be unable to adequately cope with the multi-tasking challenges that are common during everyday walking. Several interventions have been proposed for reducing the impact of DT on gait in PD [17-20]; it would, in the future, be of interest to examine if these are effective in post-DBS patients.

## Abbreviations

DBS: Deep brain stimulation; PD: Parkinson’s disease; DT: Dual task.

## Competing interests

The authors report no competing interests.

## Authors’ contributions

ESF, ZI and JMH contributed to the conception of the study and study design. ESF collected the data. AW contributed to data analyses. All authors were involved in drafting the manuscript and revising it critically for important intellectual content. All authors read and approved the final manuscript.
